# Multimodal transistors as ReLU activation functions in physical neural network classifiers

**DOI:** 10.1038/s41598-021-04614-9

**Published:** 2022-01-13

**Authors:** Isin Surekcigil Pesch, Eva Bestelink, Olivier de Sagazan, Adnan Mehonic, Radu A. Sporea

**Affiliations:** 1 Advanced Technology Institute, Department of Electrical and Electronic Engineering, University of Surrey, Guildford, GU2 7XH UK; 2 IETR-DMM-UMR6164, University of Rennes, Rennes, France; 3 Department of Electronic and Electrical Engineering, University College London, London, WC1E 6BT UK

**Keywords:** Electronic and spintronic devices, Electrical and electronic engineering, Information technology, Computer science

## Abstract

Artificial neural networks (ANNs) providing sophisticated, power-efficient classification are finding their way into thin-film electronics. Thin-film technologies require robust, layout-efficient devices with facile manufacturability. Here, we show how the multimodal transistor’s (MMT’s) transfer characteristic, with linear dependence in saturation, replicates the rectified linear unit (ReLU) activation function of convolutional ANNs (CNNs). Using MATLAB, we evaluate CNN performance using systematically distorted ReLU functions, then substitute measured and simulated MMT transfer characteristics as proxies for ReLU. High classification accuracy is maintained, despite large variations in geometrical and electrical parameters, as CNNs use the same activation functions for training and classification.

## Introduction

Current systems using CMOS, digital technologies with von Neumann architectures, are not best suited to support a massive increase in computing power demands driven by AI development^1–3^. Unconventional and analog computation approaches have emerged as an appealing alternative to CMOS and digital systems, due to the promise of increased energy efficiency and reduced circuit complexity^4–11^.

In contrast with mature ULSI CMOS technologies, thin-film, large-area circuits have numerous challenges, limiting the success of complex circuits realized at reasonable cost^12,13^. The main challenge restricting cost-effective development is the thin-film transistor (TFT), a device that comprises the backbone of many large area electronics (LAE). TFT drain current is severely prone electrode misalignments, which occur during manufacturing, resulting in a high degree of device-to-device nonuniformity. While uniformity of operation is a requirement for array-based LAE^12^, this has not limited the interest of exploring TFTs in edge processing alongside other thin-film architectures^3,14–16^. In this context, analog implementations of signal processing functions are particularly attractive, especially if the TFTs utilized are energy-efficient, as well as robust against variations during manufacturing and operation^12^. Moreover, complex circuit functions that can be performed in a compact, energy efficient footprint could further complement already attractive edge computing strategies, such as memristive neural networks^3,15^. Most common approaches to accelerate deep learning use novel nanoelectronic technologies to implement weights (synapses) in artificial neural networks^17^. Comparably much less attention has been given to efficient implementations of activation functions, which could present significant design and efficiency challenges when implemented with conventional CMOS. Such exceptionally efficient decision and classification circuits would be of great functional, economic and social benefit when included in thin-film edge processing units as part of e.g. multi-sensor, distributed or wearable electronics^18^.

**Figure 1:**
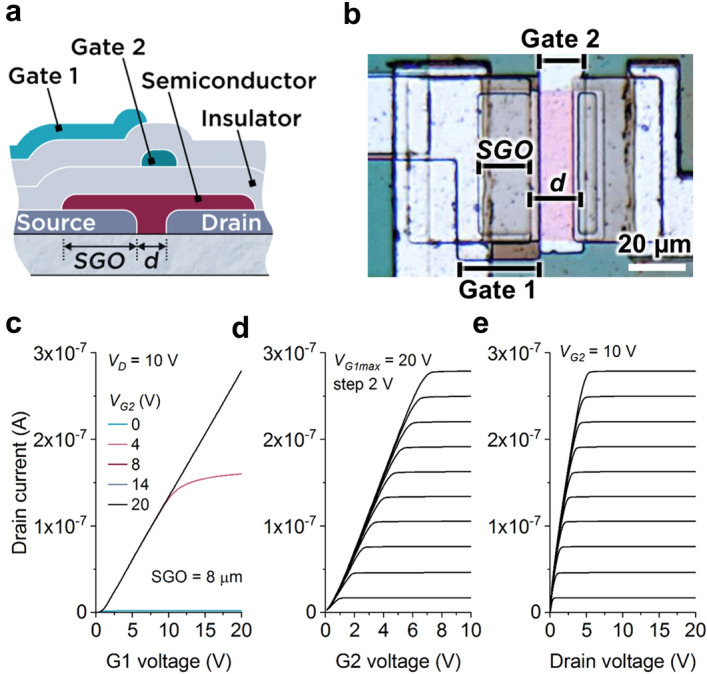
The multimodal transistor (MMT). (**a**) Illustrative cross-section and (**b**) optical micrograph of a microcrystalline silicon (µ-Si) multimodal transistor (MMT). Charge dynamics in the source-gate overlap (*SGO*) and source-drain separation (*d*) regions are controlled by the current control gate (Gate 1), and channel gate (Gate 2), respectively^19^. (**c**) Simulated amorphous silicon (a-Si) MMT transfer characteristics showing Gate 1 (G1) sets drain current magnitude, while Gate 2 (G2) allows or blocks its flow without influencing its magnitude. (**d**) Simulated transfer characteristics for G2 further demonstrating that G2 does not influence charge injection processes and thus flatten once the channel is fully accumulated. (**e**) Output characteristics showing low voltage saturation with high output impedance, expected from contact-controlled devices.

The multimodal transistor (MMT)^19^ (Fig. 1a, b) is a TFT with superior functionality, robustness and energy efficiency, especially in analog and mixed-signal applications. Notably, it can be designed with a linear dependence between input voltage and output current even when operating in saturation^19^, making it highly suited to operation as a rectified linear unit (ReLU)^20^, as this function is immediately achievable^19^. The ReLU function, defined as *max(0, x),* where *x* is the input variable, is one of the most used activation functions (AFs) in artificial neural networks (ANNs).

The performance of ANN analog accelerators (physical NNs) depends on not only the quality of the training and the precision of physical analog weights (e.g. memristors)^7^, but also, to some extent, on the accuracy of the AF. Typically, AFs are implemented with operational amplifiers^10^, which could limit the scaling perspectives. Moreover, while AFs are an important part of the neural network structure, they can also be applied to the gradient during the training process^21^. Thus, implementation of a robust AF with a single micro/nano-scale device would be highly beneficial for further development of ANN accelerators based on non-CMOS analog devices and in-memory computing concepts.

**Figure 2:**
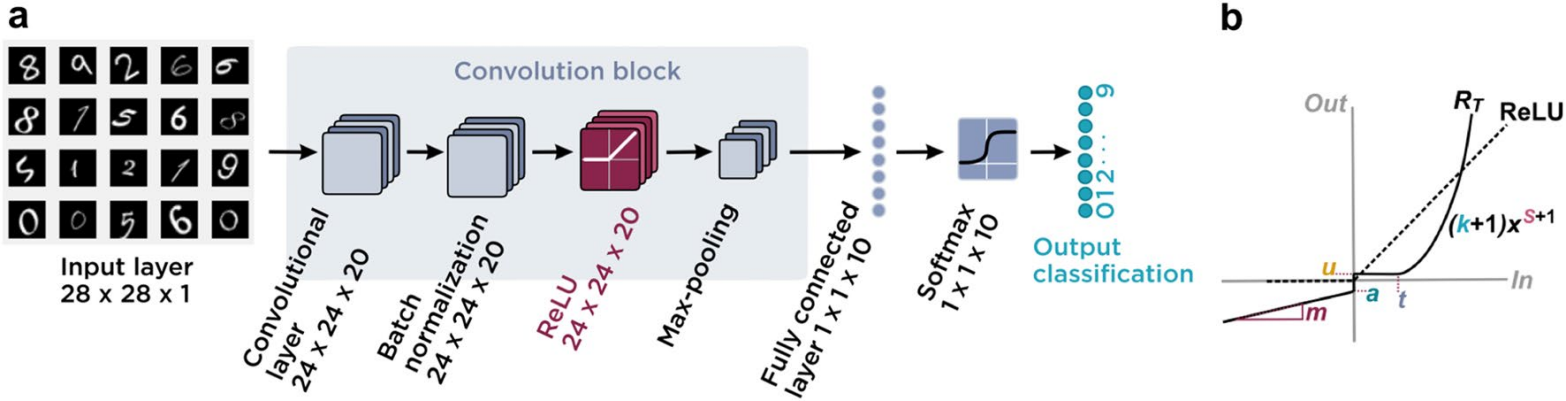
Convolutional neural network (CNN) architecture and ReLU parameter fitting. (**a**) Schematic representation of the CNN architecture and its input dataset (shown here as a screenshot of a subset of the MATLAB input data—see “Methods”). The activation functions studied are included in the ReLU layer. (**b**) Graphical representation of the parametrized activation function, “total distorted ReLU” (*R*_*T*_), used in the ReLU layer. Distortion was introduced via parameters responsible for: scaling (*k*), vertical translation (*u*), reverse leakage (*a*, *m*), horizontal translation or threshold (*t*), and polynomial behavior (*s*).

Here, we investigate the practicality of using the MMT’s transfer characteristic as a viable ReLU AF for future thin-film ANNs with high classification accuracy, despite relatively large process variations expected in such technologies. Using MATLAB, we simulate a convolutional neural network^20^ (CNN, Fig. 2a) operating with distortion parameters extrapolated from measured microcrystalline silicon (µ-Si) and simulated amorphous silicon (a-Si) MMT transfer curves as ReLU layer AFs (Fig. 2b), in comparison with the performance of MATLAB’s built-in ReLU AF.

## Multimodal transistor operation

Unlike other transistors, where a gate electrode in the channel region is responsible for controlling both charge injection and switching functions, the MMT uses the properties of a reverse-biased energy barrier at the source contact to separate these operational features^19^. Gate 1 (G1), which overlaps the source, solely controls the magnitude of charge injection in the source-G1 overlap region (SGO). Hence, the G1 transfer characteristic (Fig. 1c) resembles that of any transistor, except the drain current dependence on G1 voltage is either exponential or linear, depending on design, rather than quadratic^19^. Gate 2 (G2) controls the channel switching without influencing the magnitude of drain current, once the channel is fully accumulated (Fig. 1d), hence the curves flatten and resemble output characteristics. The output characteristics themselves are also flat (Fig. 1e), however, this is due to the nature of the energy barrier at the source contact controlling the charge injection process^19,22,23^. As long as the semiconductor is thin enough to be completely depleted at the source edge by the drain bias, the device will pinch-off at the source and very low saturation voltages *V*_*DSAT*_ can be achieved as per Eq. (1)^22,24^:

1 $${V_{DSAT}} = \left( {{V_{G1}} - {V_{th1}}} \right)\left( {\frac{{C_i}}{{{C_i} + {C_s}}}} \right) + K$$

where *C*_*i*_ and *C*_*s*_ are the gate insulator and depleted semiconductor capacitances per unit area, and *K* is the drain voltage required to deplete the charges in the accumulation layer at the insulator interface.

The choice of layer geometry and material properties will govern the nature of drain current dependence^19,25^. For high gain devices with exponential drain current dependence, the capacitance divider should yield a ratio smaller than 0.1. But in this work, some of the gain and *V*_*DSAT*_ is traded-off for constant transconductance^19^. This ability to produce a linear dependence of output on input can be useful for compact analog circuit design, such as digital-to-analog conversion^19,26^, but as the device naturally replicates the ReLU activation function, the MMT can form a useful tool in the design kit for emerging neural network implementations^19^, particularly for low-cost large area electronics.

## Results

**Figure 3:**
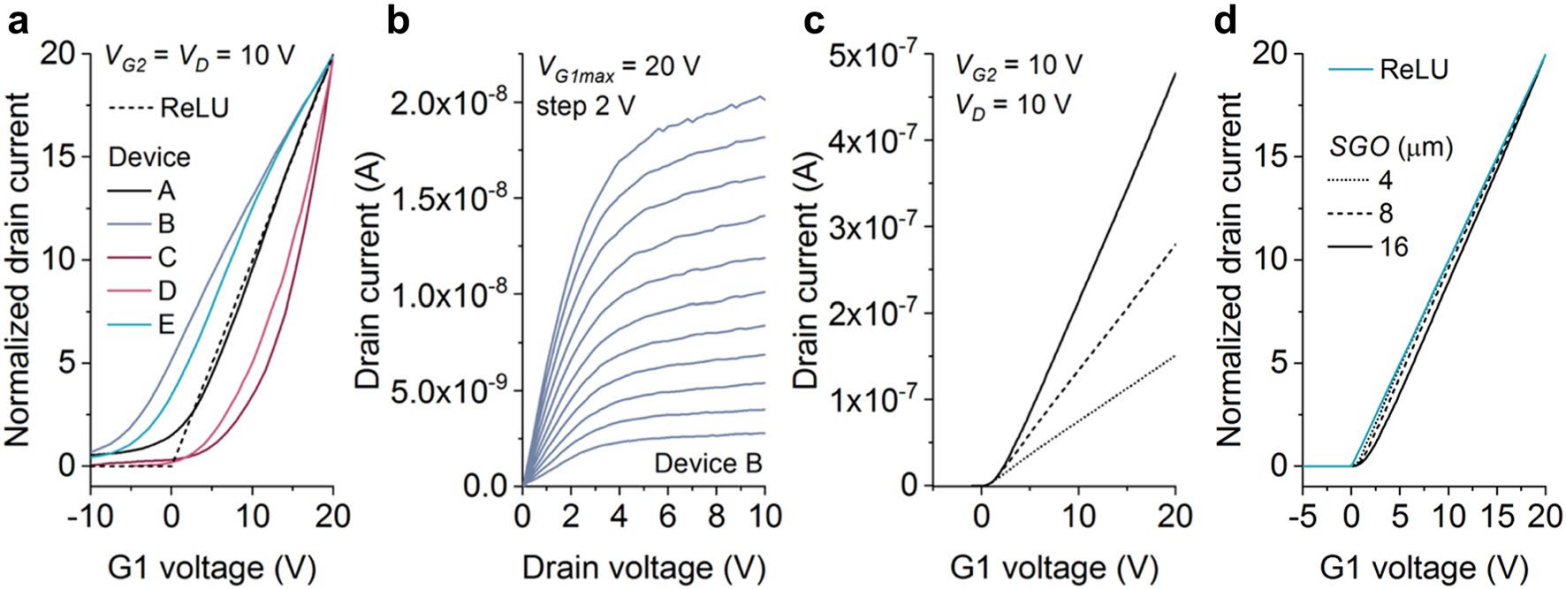
MMT characteristics. (**a**) Normalized transfer characteristics of several µ-Si MMTs showing deviation from the ReLU function. Device A (see the “Methods” section) demonstrates directly proportional dependence of drain current on G1 voltage. See Ref.^19^ for G2 transfer curves. (**b**) Measured output curves indicating low saturation voltage. For device geometries, see the “Methods” section. (**c**) Simulated transfer characteristics for a-Si MMTs with different source-G1 overlap (*SGO*). (**d**) Normalized curves to illustrate their deviation from the ReLU function.

MMT electrical measurements (Fig. 3a, b) show typical contact-controlled transistor behavior^19,22,23,27^, with low-voltage saturation (Fig. 3b). Most devices demonstrate constant transconductance *g*_*m*_ = d*I*_*D*_/d*V*_*G1*_ over a significant range of the G1 transfer characteristics (Fig. 3a), while operating in saturation. This is in contrast with the usual constant *g*_*m*_ obtained in conventional field-effect transistors exclusively in the linear region of operation. Several of the transfer curves used as practical ReLU implementations in the subsequent analysis are displayed in Fig. 3a.

TCAD simulations (Fig. 3c, d) confirm that the MMT drain current can be made directly proportional to G1 voltage^19^, with correct design. Should the off-current of such devices be many orders of magnitude lower than the on-current, the transfer curve would practically match the ReLU definition. Here, we consider several device geometries, source contact work functions, electron mobility values, and temperatures, which distort the MMT transfer curve away from the ideal ReLU shape (Fig. 2b). We modelled the deviation by assigning suitable values to the fitting parameters in Eq. (2).

2 $${R_T} = {\lambda_u}u + \begin{array}{*{20}{l}} {\left\{ {\begin{array}{*{20}{l}} {\left( {{\lambda_m}m} \right)x - {\lambda_a}a} \\ 0 \\ {\left( {{\lambda_k}k + 1} \right){{\left( {x - {\lambda_t}t} \right)}^{{\lambda_s}s + 1}}} \end{array}} \right\}}&{\begin{array}{*{20}{l}} {x < 0} \\ {0 < x < t{\lambda_t}} \\ {x > t{\lambda_t}} \end{array}} \end{array}$$

The distortion was introduced by tuning the contribution of individual parameters (Fig. 2b) responsible for scaling (*k*), vertical translation (*u*), reverse leakage (*a*, *m*), horizontal translation or threshold (*t*), and polynomial behavior (*s*) through multiplication with respective distortion factors *λ* (a number between 0 and 1). The parameter values considered for training were larger than any realistic distortion expected from practical MMTs, to amplify and discriminate the effects.

As such, the CNN-based experiments were divided into three parts, which differ only in choice of activation function. Here, the objective was not to optimize network accuracy, but to investigate how accuracy varies with device non-idealities.

Firstly, the accuracy of the network was benchmarked using the default MATLAB ReLU layer, after which, distortions were artificially introduced to emulate possible non-idealities of fabricated MMT transfer characteristics by replacing the default MATLAB ReLU function with a parametrized representation (Fig. 2b and Eq. (2), where *R*_*T*_ is the total distortion introduced into the ReLU). Table 1 lists the maximum value for each distortion parameter and the average accuracy over five classification runs, in which each parameter was enabled individually (respective λ factor equaling 1 in Eq. (2). The network was trained for all the combinations of the six parameters in Eq. (2), and the results are shown in Supplementary Table S1.

**Table 1:** Effect of varying each distortion parameter individually to its maximum value on network accuracy.

Distortion parameter and maximum value	CNN accuracy	
	Average	SD
*a* = 0.2	0.987	0.00181
*m* = 1	0.747	0.00925
*s* = 1	0.982	0.00153
*t* = 1	0.974	0.00864
*u* = 0.2	0.935	0.02187
*k* = 3	0.273	0.15191
ReLU reference	0.986	0.00236

Secondly, the parameters of Eq. (2) were fitted to the measured MMT transfer curves of Fig. 3a by selecting best-fit values for the parameters in Eq. (2). Results are shown in Table 2. Device A, which is the closest approximation of the ideal ReLU function, produces the highest accuracy. Network performance drops minutely when a negative threshold exists (Devices B and D) and deteriorates noticeably for devices with a sharper than quadratic increase (*s* > 1) of drain current with G1 voltage (Devices C and E). This is physically plausible, as MMT current can be designed to vary exponentially with G1 voltage, as the field-dependent reverse-bias current of a Schottky diode^19^.

**Table 2:** Fitting parameter values and obtained network accuracy for five measured devices.

Dev	Distortion parameter						CNN accuracy	
	*a*	*m*	*s*	*t*	*u*	*k*	Avg	SD
A	0.020	0.025	0.000	0.050	0.070	0.000	0.9848	0.0033
B	0.010	0.110	0.050	− 0.300	0.090	− 0.290	0.9778	0.0028
C	0.000	0.043	1.450	0.088	0.010	0.080	0.9462	0.0250
D	0.035	0.070	0.250	− 0.240	0.090	− 0.230	0.9817	0.0019
E	0.003	0.005	1.200	− 0.010	0.005	− 0.007	0.9600	0.0072

Finally, simulated data (Fig. 3c) produced the results as per Table 3, again based on best-fit values of the parameters in Eq. (2). We observe that all simulation conditions lead to very high network accuracy. This is most likely due to the fact that the effect of changing individual parameter values, e.g. mobility or insulator thickness, largely manifests as a scaling factor rather than a significant distortion of the characteristics (see, for example, Fig. 3d).

**Table 3:** Fitting parameter values and network accuracy for simulated devices in which one design parameter varies; *a*, *u* and *m* are always zero (see complete data in Supplementary Table S1).

Simulation parameter and value		Distortion parameter			CNN accuracy	
		*s*	*t*	*k*	Avg	SD
*SGO* (µm)	1	0.000	0.000	0.000	0.9868	0.0029
	4	0.000	0.025	0.030	0.9866	0.0020
	8	0.000	0.030	0.010	0.9850	0.0010
	16	0.030	0.043	0.015	0.9891	0.0013
*t*_*s*_ (nm)	20	0.000	0.043	0.030	0.9873	0.0031
	40	0.000	0.010	0.000	0.9859	0.0027
	80	0.000	0.010	0.000	0.9846	0.0013
*t*_*i*_ (nm)	20	0.000	0.000	0.000	0.9847	0.0012
	40	0.000	0.020	0.008	0.9870	0.0034
	80	0.010	0.043	0.009	0.9854	0.0024
*T* (K)	300	0.000	0.010	0.000	0.9871	0.0024
	320	0.000	0.025	0.008	0.9868	0.0027
	340	0.070	0.025	-0.008	0.9849	0.0020
*µ*_*n*_ (cm^2^V^-1^ s^-1^)	0.2	0.000	0.000	0.000	0.9842	0.0022
	2	0.000	0.000	0.000	0.9841	0.0016
	20	0.000	0.010	0.000	0.9857	0.0024
	200	0.010	0.045	0.030	0.9851	0.0041
*WF* (eV)	4.62	0.000	0.049	0.045	0.9871	0.0024
	4.67	0.000	0.020	0.020	0.9868	0.0027
	4.72	0.000	0.008	0.010	0.9849	0.0020

## Discussion

From Tables 2, 3 and S1, it is evident that MMT-based realizations of the ReLU layer contributes to high-accuracy classification. Practical implementations will be prone to device-to-device variations, which may be mitigated by training circuits individually to account for variability. The more convenient approach of training the network at the design phase needs to take into account practical variations, which create large absolute deviations in electrical characteristics. For example, a registration error of several microns in *SGO* changes *k, s* and *t* minimally, whereas changes in carrier mobility or operating temperature leads to unacceptably large variations of *k.*

From a functional standpoint, the channel gate (G2) and its independent control of current transport could bring additional benefits in unconventional intra-layer and inter-layer connectivity for compact implementation of classification functions.

## Conclusion

Using measured and simulated transistor data, we have shown that well-designed multimodal transistors could operate robustly as ReLU-type activations in artificial neural networks, achieving practically identical classification accuracy as pure ReLU implementations, such as the built-in MATLAB AF. The results confirm the potential of MMT devices for thin-film decision and classification circuits integrated with distributed or disposable multi-parameter sensors. Applications in wellbeing, health, environmental monitoring and smart agriculture abound.

In this initial analysis we have trained the neural network directly with the respective MMT transfer curves. On the way to full implementation, the study will continue with more computationally challenging situations, which consider device-to-device and operating variations in MMT electrical characteristics. It is expected that by closely matching the ReLU function, MMTs could provide a robust implementation of neural network activation functions, able to maintain high classification accuracy despite variability.

## Methods

### Device fabrication and characterization

Prototype bottom gate MMTs (Fig. 1b) were fabricated at low temperature using mainly ICP-CVD techniques (Corial 210-D), performing both SiO_2_ and µ–Si layers below 180 °C. The process began with deposition of the current control gate (Gate 1 or G1) in Al (Device A) or polysilicon (Devices B-E). A 100 nm SiO_2_ gate insulator was deposited before the Al channel control gate (Gate 2 or G2), which was followed by a second 100 nm SiO_2_ insulator. 40 nm µ-Si was also deposited by the same ICP-CVD reactor, followed by 20 nm SiO_2_ field plate oxide, which was patterned and etched to open contact windows for Cr source metal deposition to form Schottky contacts. See Ref.^19^ for full process details.

MMTs were electrically characterized on a Wentworth probe station connected to a B2902A source/measure unit. The transistor’s source was grounded. An additional Weir 413D power supply unit was used to provide constant 10 V on G2. MMTs with different geometries (source-G1 overlap and source-drain separation), identified as (*SGO*/*d*), were measured. Device A 54 µm/18 µm; Device B 18 µm/6 µm; Devices C and D 6 µm/6 µm; and Device E 18 µm/2 µm.

### Device simulation

MMT simulation with Silvaco Atlas v.5.24.1.R used default material parameters for intrinsic a-Si and SiO_2_.

Starting from a reference device with a source work function *WF* = 4.67 eV (to create the required Schottky barrier), source-G1 overlap *SGO* = 4 µm, semiconductor and insulator thicknesses *t*_*s*_ = *t*_*i*_ = 40 nm, respectively, electron mobility parameter *µ*_*n*_ = 20 cm^2^V^-1^ s^-1^ and default defect distribution, at temperature *T* = 300 K, we changed one of the aforementioned quantities (*WF*, *SGO*, *t*_*s*_, *t*_*i*_, *µ*_*n*_, *T*) in an exaggerated fashion to reveal variations in characteristics. As drain current is not modulated by the channel region, source-drain separation was kept constant at *d* = 4 µm. G2 was self-aligned to the drain. See Ref.^19^ for detailed simulation and structure parameters.

### Artificial neural network simulation

A CNN with fully connected layers was created using the MATLAB deep learning toolbox and trained with the standard recommended setup^28^ (Fig. 2a). The network contained 28 × 28 × 1 image input, 24 × 24 × 20 convolution 2D, 24 × 24 × 20 batch normalization, 24 × 24 × 20 ReLU, 1 × 1 × 10 fully connected, 1 × 1 × 10 softmax, and 1 × 1 × 10 classification layers (Fig. 2a). The CNN was trained to recognize handwritten numbers provided in the MATLAB Digits dataset^28^, containing 10,000 images from the Modified National Institute of Standards and Technology (MNIST)^29^ dataset.

As the network had to be trained numerous times, relatively simple training options were used: 0.01 initial learning rate; 232 iterations; 4 max epochs; and included the algorithm stochastic gradient descent with momentum optimizer. The CNN contained a ReLU layer, which was used by the optimizer algorithm to adjust weights during training (Fig. 2a). The same activation function was used across training and classification tasks.

## Data availability

Data and materials can be obtained via the corresponding author, on request.

## Supplementary Information

Supplementary Information.
