# Supplementary material for: Multimodal transistors as ReLU activation functions in physical neural network classifiers
Source: Sci Rep. 2022 Jan 13;12:670. doi: 10.1038/s41598-021-04614-9 (PMC8758690; doi:10.1038/s41598-021-04614-9)
Supplement: Supplementary file 1 — Supplementary Information. [file 41598_2021_4614_MOESM1_ESM.pdf]

SUPPLEMENTARY MATERIAL:

Multimodal transistors as ReLU activation functions in physical neural network classifiers

Isin Surekcigil Pesch, Eva Bestelink, Olivier de Sagazan, Adnan Mehonic & Radu A. Sporea

Table S1. **Multi-parameter simulation of network accuracy for all combinations of distortion parameter variation.** The legend below shows the simplified notation with roman capitals of the variation of each distortion parameter. In the main table, the presence of the capital letter shows that the respective distortion has been applied. Also shown in the legend is the statistical significance of the effect of enabling each of the distortion parameters on the network accuracy values. Parameters *a*, *m* and *u* (see Fig. 2b) appear not to influence the network accuracy. This is discussed in the main text and represents the reason for omitting them from Table III, which synthesizes the important effects.

Significance	λa	λm	λs	λt	λu	λk
	A	B	C	D	E	F
99.9%	No	No	Yes	Yes	No	Yes

Simulation	λ factor comb.	CNN Accuracy			Simulation	λ factor comb.	CNN Accuracy		
		Avg.	Variance	Stdev.			Avg.	Variance	Stdev.
**1**	0	0.9864	0.0000	0.0026	**33**	F	0.2726	0.0288	0.1698
**2**	A	0.9867	0.0000	0.0020	**34**	AF	0.7369	0.0131	0.1144
**3**	B	0.7467	0.0001	0.0103	**35**	BF	0.8399	0.0018	0.0424
**4**	AB	0.7482	0.0003	0.0177	**36**	ABF	0.8306	0.0006	0.0244
**5**	C	0.9818	0.0000	0.0017	**37**	CF	0.9794	0.0000	0.0055
**6**	AC	0.9817	0.0000	0.0054	**38**	ACF	0.9818	0.0001	0.0085
**7**	BC	0.9359	0.0000	0.0062	**39**	BCF	0.9570	0.0001	0.0095
**8**	ABC	0.9134	0.0000	0.0064	**40**	ABCF	0.9534	0.0001	0.0082
**9**	D	0.9742	0.0001	0.0097	**41**	DF	0.1000	0.0000	0.0000
**10**	AD	0.9812	0.0000	0.0061	**42**	ADF	0.0800	0.0020	0.0447
**11**	BD	0.8883	0.0003	0.0170	**43**	BDF	0.0000	0.0000	0.0000
**12**	ABD	0.8970	0.0002	0.0154	**44**	ABDF	0.0000	0.0000	0.0000
**13**	CD	0.9771	0.0000	0.0047	**45**	CDF	0.2554	0.1193	0.3453
**14**	ACD	0.9741	0.0000	0.0047	**46**	ACDF	0.5996	0.0218	0.1478
**15**	BCD	0.9714	0.0000	0.0043	**47**	BCDF	0.0000	0.0000	0.0000
**16**	ABCD	0.9716	0.0000	0.0048	**48**	ABCDF	0.0000	0.0000	0.0000
**17**	E	0.9348	0.0006	0.0244	**49**	EF	0.1634	0.0078	0.0884
**18**	AE	0.9786	0.0000	0.0058	**50**	AEF	0.2620	0.0172	0.1312
**19**	BE	0.7309	0.0003	0.0161	**51**	BEF	0.7414	0.0054	0.0736
**20**	ABE	0.7641	0.0002	0.0134	**52**	ABEF	0.8427	0.0020	0.0449
**21**	CE	0.9818	0.0000	0.0039	**53**	CEF	0.9072	0.0025	0.0503
**22**	ACE	0.9834	0.0000	0.0021	**54**	ACEF	0.9346	0.0038	0.0613
**23**	BCE	0.9406	0.0001	0.0086	**55**	BCEF	0.9393	0.0001	0.0099
**24**	ABCE	0.9128	0.0001	0.0083	**56**	ABCEF	0.9434	0.0001	0.0077
**25**	DE	0.8376	0.0155	0.1247	**57**	DEF	0.1000	0.0000	0.0000
**26**	ADE	0.9534	0.0004	0.0212	**58**	ADEF	0.1001	0.0000	0.0002
**27**	BDE	0.8780	0.0006	0.0252	**59**	BDEF	0.0000	0.0000	0.0000
**28**	ABDE	0.8144	0.0042	0.0650	**60**	ABDEF	0.0000	0.0000	0.0000
**29**	CDE	0.9714	0.0001	0.0088	**61**	CDEF	0.1026	0.0000	0.0057
**30**	ACDE	0.9680	0.0000	0.0070	**62**	ACDEF	0.5590	0.0007	0.0260
**31**	BCDE	0.9675	0.0000	0.0046	**63**	BCDEF	0.1499	0.1124	0.3352
**32**	ABCDE	0.9666	0.0000	0.0037	**64**	ABCDF	0.0000	0.0000	0.0000
